# Supplementary material for: Perinuclear Anti-Neutrophil Cytoplasmic Antibodies (pANCA) Impair Neutrophil Candidacidal Activity and Are Increased in the Cellular Fraction of Vaginal Samples from Women with Vulvovaginal Candidiasis
Source: J Fungi (Basel). 2020 Oct 16;6(4):225. doi: 10.3390/jof6040225 (PMC7712103; doi:10.3390/jof6040225)
Supplement: Supplementary file 1 [file jof-06-00225-s001.pdf]

Supplemental Figures and Tables

Table S1. Clinical features of the enrolled patients, immunological parameters (CAGTA IgA, ASCA, pANCA and S100A8) of the vaginal fluids and protein extracts, and subsets of tests matched to patient samples. Data points in red indicate that the values are below the detection limit of the kit. pANCA data from PEs are expressed in pg/µg of total protein. Yellow coded: first group of samples used for antibodies and S100A8 assessment only in VF. Blue coded: second group of samples, some of which were used for different determinations (RNA extraction from cellular fraction and antibody assessment in parallel in VF and PE). Green coded: clinical samples from patients first described in Pericolini et al. 2018 (1) and further analyzed here. Abbreviations: n.a.: data not available; N: no; Y: yes.

					Patient status, microbiology and pH					ASCA in VF (U/ml)		pANCA IgG			Participant subsets for different analyses				
Clinical condition	PMN Infiltration	Patient ID	Fungal species	CAGTA IgA	Bacteria	Lactobacilli	Co-infection	Pregnancy	pH	IgA	IgG	VF (pg/ml)	PE (pg/µg)	S100A8 (pg/ml)	VF for ASCA, pANCA and CAGTA	VF for S100A8	Cellular fraction stored in Trizol for RNA extraction and gene expression analysis	PE from Cellular fraction analyzed for ASCA and pANCA	Studied in Pericolini et al. (1)
VVC	High or Massive (HI)	SP-4248	*C. albicans*	+	+	+	GBS	N	4	0	0	169.2	n.a.	n.a.					
		SP-0877	*C. albicans*	+	+	+	none	N	5	0	0	0	n.a.	n.a.					
		SP-2464	*C. albicans*	+	+	+	none	N	4	0	0	20.7	n.a.	448.1					
		SP-1980	*C. albicans*	-	+	+	none	N	4	0	0	24.8	n.a.	394.3					
		SP-1892	*C. albicans*	+	+	+	none	N	n.a.	0	0	81.2	n.a.	444.9					
		SP-4848	*C. albicans*	+	n.a.	+	n.a.	N	4	0	0	1489.6	n.a.	120.1					
		SP-1074	*C. albicans*	+	n.a.	-	n.a.	N	5	0	0	567.7	n.a.	275.2					
		SP-4581	*C. albicans*	-	n.a.	+	n.a.	N	5	0	0	1356.7	n.a.	145.6					
		SP-3744	*C. albicans*	-	n.a.	+	n.a.	N	n.a.	0	0	747.7	n.a.	157.1					
		SP-7001	*C. albicans*	-	+	-	*G. vaginalis*	Y	5	1.3	0	117.8	8.76	362.9					
		SP-4976	*C. albicans*	-	+	+	none	Y	n.a.	1.2	0	141.2	7.24	431.6					
		SP-5980	*C. albicans*	+	+	+	GBS	Y	n.a.	1.1	0	191.1	7.65	81.7					
		SP-1523	*C. albicans*	-	+	+	none	N	4	0	0	175.9	n.a.	n.a.					
		SP-2300	*C. albicans*	+	+	-	none	Y	5	0	0	212.3	n.a.	90.3					
		SP-2009	*C. albicans*	+	+	+	none	Y	n.a.	4.7	0	77.3	n.a.	441.4					
		SP-4900	*C. albicans*	+	n.a.	+	n.a.	Y	4	4.73	0	2016.7	n.a.	247.7					
		SP-7753	*C. albicans*	+	+	+	GBS	N	4	0.5	0	837.0	7.82	n.a.					
		SP-1256	*C. albicans*	+	+	-	*G. vaginalis*	N	5	2.1	0	1242	7.11	n.a.					
		SP-4914	*C. albicans*	+	+	-	GBS + *G. vaginalis*	N	5	1.3	0	1722	7.84	n.a.					
		SP-6195	*C. albicans*	+	n.a.	+	n.a.	Y	n.a.	0	0	1600.9	n.a.	233.7					
		SP-7160	*C. albicans*	+	n.a.	+	n.a.	Y	n.a.	0	0	1592.5	n.a.	173.1					
		SP-7983	*C. albicans*	+	n.a.	+	n.a.	Y	5	0	0	2094.7	n.a.	286.5					
		SP-9182	*C. albicans*	+	n.a.	+	n.a.	Y	5	0	0	563.1	n.a.	288.42					
		SP-9654	*C. albicans*	+	n.a.	+	n.a.	Y	5	0	0	712.2	n.a.	388.2					
		SP-3457	*C. albicans*	-	n.a.	+	n.a.	Y	n.a.	0	0	1486.8	n.a.	103.6					
		SP-8566	*C. albicans*	+	n.a.	+	n.a.	Y	n.a.	0	0	731.1	n.a.	158.8					
		SP-3544	*C. albicans*	-	n.a.	+	n.a.	Y	5	0	0	880.5	n.a.	156.6					
		SP-5811	*C. albicans*	-	n.a.	+	n.a.	Y	5	0	0	2318.4	n.a.	308.1					
Colonized	Low or None (LI)	SP-8387	*C. albicans*	-	+	+	none	N	n.a.	0	0	81.22	5.72	n.a.					
		SP-5231	*C. albicans*	-	+	+	GBS	N	4	0	0.5	0	5.08	348.7					
		SP-4314	*C. albicans*	-	+	+	none	N	4	0.2	0	332.4	n.a.	403.6					
		SP-1125	*C. albicans*	+	+	-	none	N	5	0.3	0	186.0	n.a.	293.6					
		SP-9246	*C. albicans*	+	+	+	none	N	4	0	0	175.9	n.a.	305.2					
		SP-4797	*C. albicans*	-	n.a.	+	n.a.	N	n.a.	0	0	1531.0	n.a.	275.9					
		SP-7228	*C. albicans*	-	n.a.	-	n.a.	N	n.a.	0	0	1640.4	n.a.	211.0					
		SP-7621	*C. albicans*	-	n.a.	+	n.a.	N	4	0	0	1035.7	n.a.	463.9					
		SP-4168	*C. albicans*	+	n.a.	+	n.a.	N	5	0	0	1043.2	n.a.	447.8					
		SP-8467	*C. albicans*	-	n.a.	-	n.a.	N	5	0	0	1924.8	n.a.	277.7					
		SP-8605	*C. albicans*	-	n.a.	+	n.a.	N	4	0	0	1116.6	n.a.	214.3					
		SP-3214	*C. albicans*	+	n.a.	+	n.a.	N	4	0	0	2013.5	n.a.	36.6					
		SP-4134	*C. albicans*	-	n.a.	+	n.a.	N	4	0	0	1295.6	n.a.	425.6					
		SP-6884	*C. albicans*	-	+	+	none	Y	4	0.2	0	51.2	6.11	n.a.					
		SP-1566	*C. albicans*	-	+	+	none	Y	4	0	0	315.7	n.a.	23.3					
		SP-6850	*C. albicans*	-	+	+	GBS	Y	4	0	0	170.5	4.53	n.a.					
		SP-6507	*C. albicans*	+	+	+	none	N	5	3.9	0	1516	8.37	n.a.					
		SP-7881	*C. albicans*	+	+	+	none	N	5	1.85	0	1549	6.86	n.a.					
		SP-5936	*C. glabrata*	+	+	+	none	N	4	0	0	169.2	5.51	430.5					
		SP-9330	*C. glabrata*	+	+	+	none	N	5	1.1	0	149.1	n.a.	305.8					
		SP-0605	*C. glabrata*	+	+	+	GBS	N	4	0	0	371.7	n.a.	479.2					
		SP-5949	*C. krusei*	+	+	+	none	N	5	0.4	0	214.7	3.49	219.4					
		SP-5194	*. guillermondii*	-	+	+	none	N	5	0	0	62.3	6.58	454.6					

Key		
	**Red numbers: values below the cut-off level of detection**	VF: vaginal fluids
	**n.a.: data not available for this sample**	PE: protein extracts

Reference:

1. Pericolini E, Perito S, Castagnoli A, Gabrielli E, Mencacci A, Blasi E, Vecchiarelli A, Wheeler RT. 2018. Epitope unmasking in vulvovaginal candidiasis is associated with hyphal growth and neutrophilic infiltration. PLoS One 13:e0201436.

Table S2. Primers used for real-time PCR analysis.

-h-MPO FWD: 5′-TTT GAC AAC CTG CAC GAT GAC-3′
-h-MPO REV: 5′-CGG TTG TGC TCC CGA AGT AA-3′
-h-CD11b FWD: 5′-GAT CCA ACC TAC GGC AGC AG-3′
-h-CD11b REV: 5′-ATC CGC CGA AAG TCA TGT GG-3′
-h-GADPH FWD: 5′-CGG ATT TGG TCG TAT TGG G-3′
-h-CD11b REV: 5′-CTC GCT CCT GGA AGA TGG-3′

Table S3. Clinical features of the healthy non-colonized enrolled women, and immunological parameters (CAGTA IgA, ASCA, pANCA and S100A8) of the VF. Data points in red indicate that the values are below the detection limit of the kit. Abbreviations: n.a.: data not available; N: no; Y: yes.

					Patient status, microbiology and pH					ASCA in VF (U/ml)		pANCA IgG		S100A8 (pg/ml)
Clinical condition	PMN Infiltration	Patient ID	Fungal species	CAGTA IgA	Bacteria	Lactobacilli	Co-infection	Pregnancy	pH	IgA	IgG	VF (pg/ml)	PE (pg/µg)	
non-Colonized	None	SP-2894	*none*	+	+	+	none	N	4	0.5	0	177.6	n.a.	422.6
		SP-6653	*none*	-	+	+	none	N	4	0.22	0	0	n.a.	205
		SP-2378	*none*	-	+	-	*G. vaginalis*	N	n.a.	1.6	0	1058.3	n.a.	434.3
		SP4652	*none*	-	n.a.	+	n.a.	N	n.a.	0	0	1770.1	n.a.	363
		SP-0995	*none*	-	n.a.	+	n.a.	N	n.a.	0	0	2069.6	n.a.	279.8
		SP-6878	*none*	-	n.a.	+	n.a.	N	4	2.3	0	0	n.a.	0
		SP-8270	*none*	-	n.a.	+	n.a.	N	n.a.	0	0	1837.8	n.a.	6.5
		SP-7261	*none*	-	+	+	none	Y	4	0	0.39	109	n.a.	418.4
		SP-8031	*none*	-	+	+	none	Y	n.a.	0	0	183.7	n.a.	63.2

Key		
	Red numbers: values below the cut-off level of detection	VF: vaginal fluids
	n.a.: data not available for this sample	PE: protein extracts

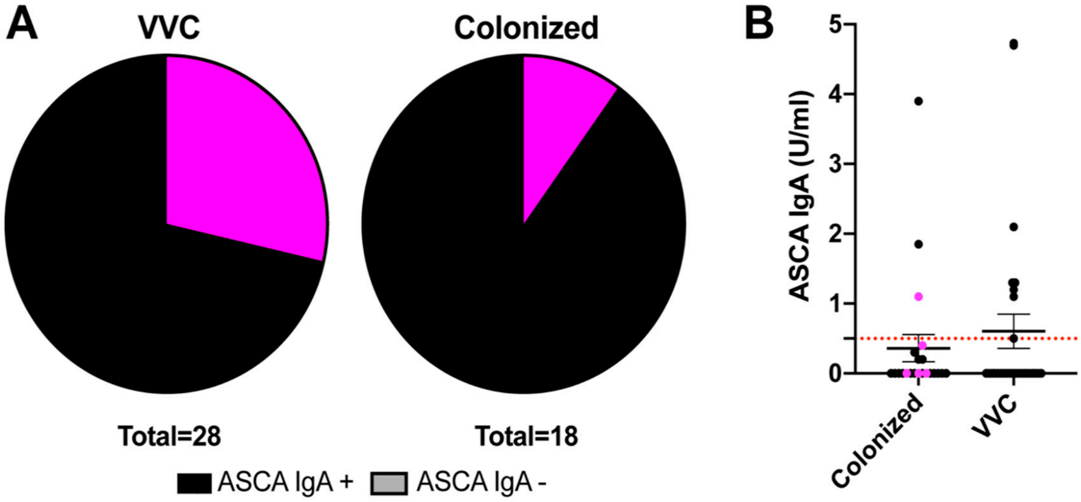

Figure S1. ASCA IgA determination. **A**: Pie charts of ASCA IgA in positive (magenta) and negative (black) samples from vaginal fluids (VF) of VVC and colonized women. **B**: Mean ± SEM of ASCA IgA (U/mL) in vaginal fluids (VF) from VVC and colonized women.

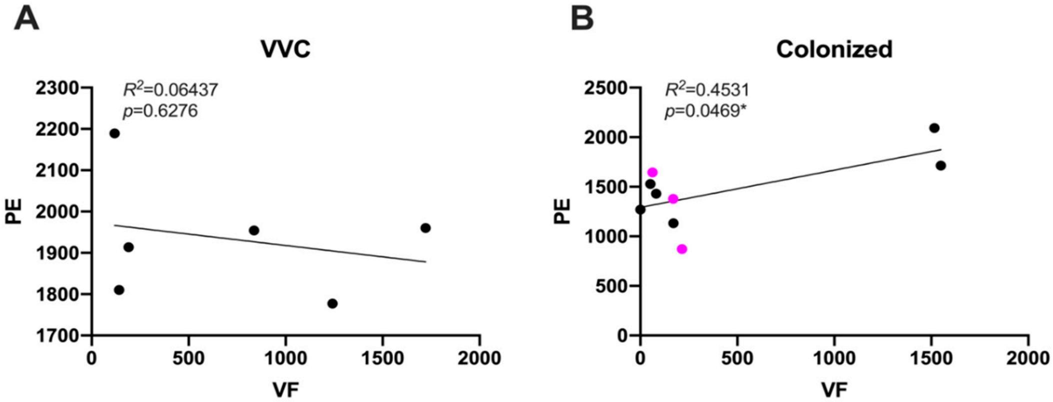

Figure S2. Pearson's correlation graphs of pANCA IgG levels in VF or PEs of VVC and colonized women. Values of pANCA IgG in VF and PEs from VVC (**A**) and colonized (**B**) women were analyzed by simple linear regression. R squared (r^2) and p values are reported in the graphs.
